# Chloride, Sodium and Calcium Intake Are Associated with Mortality and Follow-Up Kidney Function in Critically Ill Patients Receiving Continuous Veno-Venous Hemodialysis—A Retrospective Study

**DOI:** 10.3390/nu15030785

**Published:** 2023-02-03

**Authors:** Tapio Hellman, Panu Uusalo, Mikko J. Järvisalo

**Affiliations:** 1 Kidney Center, Turku University Hospital, University of Turku, Hämeentie 11, P.O. Box 52, 20521 Turku, Finland; 2 Department of Anaesthesiology and Intensive Care, Turku University Hospital, University of Turku, Hämeentie 11, P.O. Box 52, 20521 Turku, Finland; 3 Perioperative Services, Intensive Care and Pain Medicine, Turku University Hospital, University of Turku, Hämeentie 11, P.O. Box 52, 20521 Turku, Finland

**Keywords:** intensive care, chloride, mortality, nutrition, kidney function

## Abstract

Background: Studies on the association between solute, nutrition and fluid intakes and mortality and later kidney function in critically ill acute kidney injury (AKI) patients receiving continuous veno-venous hemodialysis (CVVHD) are scarce. Methods: Altogether, 471 consecutive critically ill AKI patients receiving CVVHD in the research intensive care unit (ICU) were recruited in this single-center, retrospective study. Results: The median age was 66 (58–74) years, and 138 (29.3%) were female. The 90-day and one-year mortalities were 221 (46.9%) and 251 (53.3%), respectively. After adjusting for age, sex, Acute Physiology and Chronic Health Evaluation II (APACHE) score, coronary artery disease, immunosuppression, ICU care duration, mechanical ventilation requirement, vasopressor requirement and study time period, the cumulative daily intake of potassium, chloride, sodium, phosphate, calcium, glucose, lipids and water was associated with one-year mortality in separate multivariable cox proportional hazards models. In a sensitivity analysis excluding patients who died within the first three days of ICU care, the daily intake of chloride (hazard ratio (HR) 1.001, confidence interval (CI) 95% 1.000–1.003, *p* = 0.032), sodium (HR 1.001, CI 95% 1.000–1.002, *p* = 0.031) and calcium (HR 1.129, CI 95% 1.025–1.243, *p* = 0.014) remained independently associated with mortality within one-year in the respective, similarly adjusted multivariable cox analyses. The cumulative daily intake of chloride, sodium, calcium and water was independently associated with the estimated glomerular filtration rate (eGFR) at 90 days follow-up in separate substantially adjusted multivariable cox proportional hazards models. Conclusion: The cumulative daily intake of chloride, sodium and calcium is associated with mortality and daily chloride, sodium, calcium and water intake is associated with follow-up eGFR in critically ill patients with CVVHD-treated AKI.

## 1. Introduction

Intense research on optimal fluid resuscitation and nutrition in critically ill patients has been ongoing in recent years. Several large studies and randomized controlled trials (RCTs) have compared the effect of administering saline versus balanced crystalloids (e.g., Ringer’s solution or Plasma-Lyte) for fluid resuscitation on patient and renal survival with inconsistent results [1,2,3,4,5,6,7]. The use of saline has been associated with diminished renal perfusion, sustained hyperchloremia and interstitial edema [8], generating a hypothesis for associated adverse patient and renal outcomes [1,4,7]. However, a large meta-analysis on the comparison of saline and balanced crystalloids did not demonstrate consistent survival benefits in relation to either solution. However, saline (chloride concentration 154 mmol/L) has shown better results in the treatment of traumatic brain injury, while patients without traumatic brain injury have benefitted more from balanced crystalloids (chloride concentration: Plasma-Lyte 98 mmol/L and Ringer 112 mmol/L) [9]. Importantly, there are no studies on the effects of chloride intake on patient or renal outcomes in critically ill acute kidney injury (AKI) patients receiving continuous renal replacement therapy (CRRT).

The early initiation of nutrition (within 48 h) and preference of the oral or enteral route over the parenteral route are recommended by the European Society for Clinical Nutrition and Metabolism (ESPEN) guidelines on nutrition for hospitalized AKI patients, as well as critically ill patients, while parenteral nutrition should only be initiated within 3–7 days in those with contraindications to oral or enteral nutrition [10,11]. In a recent meta-analysis, enteral nutrition was not associated with a survival benefit compared to parenteral nutrition in critically ill patients, but decreased the risk of infectious complications and the length of the hospital stay [12]. As renal replacement therapy (RRT) in critically ill patients can be associated with catabolic protein metabolism, a higher protein intake is recommended, while no recommendations are given in relation to lipid or glucose/carbohydrate intake in these patients [10]. Furthermore, no specific guidelines on the nutrition of critically ill patients receiving CRRT exist.

Thus, we sought to study the association between cumulative daily solute, nutrition and fluid intake in critically ill continuous veno-venous hemodialysis (CVVHD) patients during intensive care unit (ICU) care based on all-cause mortality and follow-up kidney function in this retrospective real-world cohort study.

## 2. Materials and Methods

The association between daily cumulative solute, nutrition and fluid intake during ICU care on mortality and kidney function was studied in this prespecified retrospective study on critically ill patients receiving CVVHD. Altogether, 493 consecutive critically ill patients treated with CVVHD as the first RRT modality in the research ICU between 1 January 2010 and 31 December 2019 were screened. All patients on maintenance dialysis (*n* = 22) were excluded yielding a study cohort of 471 patients.

Data on patient demographics and baseline medical history were manually collected from the electronic patient records of the research hospital by the researchers. Extensive daily cumulative data on received solutes, nutrition and fluids and general ICU care specifics were extracted from the clinical software of the research ICU. Because specific patient data on the administered commercial preparations or applied electrolyte concentrates were not available, the data on the received solutes, nutrition and fluids were extracted as total cumulative measures per ICU care period for each patient from the patient records.

Estimated glomerular filtration rate (eGFR) was calculated using the Chronic kidney disease—Epidemiology collaboration (CKD-EPI) algorithm. Baseline eGFR was denoted as the most recent eGFR measurement within one year prior to ICU admission. Immunosuppression was defined as ongoing immunosuppressive medication: corticosteroids (methylprednisolone dose (or equivalent dose) exceeding 10 mg/day), calcineurin inhibitors, mycophenolate mofetil, azathioprine, immunosuppressive monoclonal antibodies used for active autoimmune disorders or cytotoxic chemotherapy for the management of solid organ or hematologic malignancies administered within one year prior to admission. The presence of sepsis was defined as a suspected infection with a Sequential Organ Failure Assessment (SOFA) score ≥2.

For the purpose of studying the changes in the intakes of solutes with the strongest associations with patient and renal outcomes over time, the study period was divided into quarters (first: January 2010–June 2012; second: July 2012–January 2015; third: February 2015–August 2017, fourth: September 2017–December 2019).

The primary outcome measure of the study was all-cause mortality within a one-year follow-up and the secondary outcome was denoted eGFR at 90 days follow-up in survivors.

CVVHD was performed according to a standard protocol employed in the research ICU. Fresenius Multifiltrate CRRT monitors and 1.80 m^2^ polysulfone hemofilter Ultraflux AV1000 or Ultraflux EmiC2 (Fresenius Medical Care Deutschland GmbH, Bad Homburg, Germany) membranes were used with the CiCa^®^ dialysate K2 and 4% trisodium citrate to achieve regional citrate anticoagulation (Fresenius Medical Care, Bad Hamburg, Germany). Blood and dialysate flow rates were adjusted to target a dialysis dose of 30 mL/kg/h according to the body weight of the patient. The CVVHD protocol remained unchanged during the study.

The latest European Society for Clinical Nutrition and Metabolism (ESPEN) guidelines on nutrition in critically ill patients were adopted and implemented in the research ICU throughout the study period. By the last quarter of the study period, the preferred resuscitation solutions were changed from normal saline or Ringer’s solution to balanced crystalloids (Ringer’s (Fresenius Kabi AG, Bad Homburg, Germany) or Plasma-Lyte (Baxter International Inc., Deerfield, IL, USA) solution). The choice of resuscitation solutions was left to the discretion of attending clinicians.

### 2.1. Ethics

The study protocol was approved by the Turku University Clinical Research Center scientific review board and the Hospital district of Southwest Finland (Reference number: T143/2019). For this retrospective, register-based non-interventional study, the Ethics Committee of the Hospital District of Southwest Finland waived the need for informed consent in terms of data collection and the analysis and publication of results. The study adhered to the Declaration of Helsinki.

### 2.2. Statistics

Categorical variables are reported with absolute and relative (percentage) frequencies and continuous variables as the median and inter-quartile range (IQR), as all continuous variables had a skewed distribution. The normality of continuous variables was tested with the Kolmogorov–Smirnov and Shapiro–Wilk tests. Categorical variables were compared with Pearson ×2 or Fisher’s exact tests and continuous variables were compared with the Mann–Whitney U test.

As the purpose of this study was to assess the outcomes related to the cumulative intake of solutes, nutrition and fluid during varying durations of ICU care, the intake measures are reported as daily total cumulative intake (total intake divided by the ICU care duration in days). The results of univariate and multivariable cox proportional hazards and linear regression analyses are reported per one unit increase in intake (e.g., per one mmol increase in the intake of chloride).

The univariate associations between tested covariates and mortality within one year were analyzed with cox proportional hazards models. The multivariable cox proportional hazards models for one-year mortality were performed by including age, sex, immunosuppression, coronary artery disease, Acute Physiology And Chronic Health Evaluation II (APACHE) score, ICU care duration in days, change in study time (in quarters), requirement for mechanical ventilation and vasopressor requirement, in addition to a single covariate of interest (e.g., daily chloride intake) in each respective model. The multivariable cox proportional hazards analysis analyzing the association between the cumulative daily intake of chloride and one-year mortality was further adjusted with baseline chloride measured at the time of admission. The survival analyses for mortality were repeated in a sensitivity analysis on a subcohort excluding patients who died within the first 3 days of ICU care. Finally, the association between one-year mortality and the cumulative intake of chloride as a categorical dichotomous variable (cut-off set at median) was analyzed in an unadjusted Kaplan–Meier time-to-event model with the use of a log-rank test.

The univariate associations between tested independent variables and eGFR at 90 days follow-up were analyzed with linear regression models in surviving patients with available eGFR data (*n* = 189). The separate multivariable regression models were adjusted with age, sex, immunosuppression, coronary artery disease, APACHE-score, ICU care duration in days, need for mechanical ventilation, vasopressor requirement and baseline eGFR as covariates. The association between the cumulative intake of chloride and eGFR at 90 days follow-up was further tested by entering baseline chloride in the adjusted multivariable linear regression model.

The relationship between hospital mortality and change in time (measured in quarters of the study period) or the cumulative daily intake of chloride was analyzed in univariate logistic regression models. The Nagelkerke R square was measured to assess the explanatory proportion of variation of the dependent variable for each covariate. Finally, both the change in time and cumulative intake of chloride were entered in a multivariable logistic regression model for hospital mortality.

The total missingness of all independent variables was 0.8%, and a complete case approach in the analyses was used.

The potential existence of multicollinearity was assessed by examining variance inflation factors.

All analyses were two-sided, and *p* < 0.05 was considered statistically significant. IBM SPSS Statistics software version 26.0 (IBM, Armonk, NY, USA) was used to perform all analyses.

## 3. Results

The baseline characteristics and ICU care specifics of the study patients are summarized in Table 1. The median age was 66 (58–74) years, 138 (29.3%) were female, 175 (37.2%) patients were surgical and 253 (53.7%) had sepsis. Hospital, 90-day, and 1-year mortality were 197 (41.8%), 221 (46.9%) and 251 (53.3%), respectively. The median eGFR at 90 days follow-up in survivors was 55.6 (37.1–86.6) mL/min/1.73 m^2^. Baseline biochemical data and cumulative daily solute, nutrition and fluid intake data are shown in Table 2.

**Figure 1:**
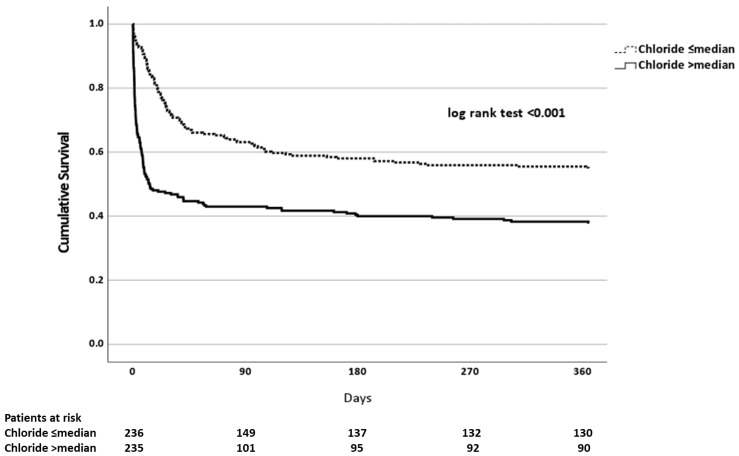
Kaplan–Meier plot with a log-rank test depicting the association between the daily cumulative intake of chloride as a dichotomous categorical variable (cut-off set at median, 192 mmol) and one-year mortality.

The associations between explored covariates and one-year mortality in univariate cox proportional hazards models are presented in Table 3. After adjusting for age, sex, APACHE-score, coronary artery disease, immunosuppression, ICU care duration in days, need for mechanical ventilation, vasopressor requirement and study time, the daily intakes of potassium (hazard ratio (HR) 0.989, confidence interval (CI) 95% 0.982–0.997, *p* = 0.005), chloride (HR 1.002, CI 95% 1.002–1.003, *p* < 0.001), sodium (HR 1.002, CI 95% 1.001–1.002, *p* < 0.001), phosphate (HR 0.941, CI 95% 0.925–0.958, *p* < 0.001), calcium (HR 1.145, CI 95% 1.115–1.177, *p* < 0.001), glucose (HR 1.003, CI 95% 1.000–1.005, *p* = 0.040), lipids (HR 0.980, CI 95% 0.969–0.992, *p* = 0.001) and water (HR 1.000, CI 95% 1.000–1.000, *p* < 0.001) were independently associated with one-year mortality in separate multivariable cox proportional hazards analyses. When the patients that perished (*n* = 84 (17.8%)) within the first 3 days of ICU care were excluded from the analyses, the daily intake of chloride (HR 1.001, CI 95% 1.000–1.003, *p* = 0.032), sodium (HR 1.001, CI 95% 1.000–1.002, *p* = 0.031) and calcium (HR 1.129, CI 95% 1.025–1.243, *p* = 0.014) remained independently associated with one-year mortality in similarly adjusted respective multivariable cox proportional hazards models. The daily intake of chloride remained independently associated with one-year mortality when the multivariable cox proportional hazards analysis was further adjusted with baseline chloride in the whole cohort (HR 1.002, CI 95% 1.002–1.003, *p* < 0.001), as well as in those that survived the first 3 days in the ICU (HR 1.001, CI 95% 1.000–1.003, *p* = 0.031). The association between daily chloride intake as a dichotomous variable and one-year mortality is visualized in Figure 1.

eGFR data were available in 189 (75.6%) patients out of the 250 survivors at 90 days follow-up. The associations between tested covariates and eGFR at 90 days follow-up in univariate linear regression models are presented in Table 3. The daily intakes of chloride (β = 0.215, CI 95% 0.026–0.119, *p* = 0.003), sodium (β = 0.253, CI 95% 0.030–0.100, *p* < 0.001), calcium (β = 0.155, CI 95% 0.783–7.020, *p* = 0.015) and water (β = 0.307, CI 95% 0.006–0.015, *p* < 0.001) were independently associated with eGFR at 90 days follow-up in separate multivariable cox proportional hazards models adjusted for age, sex, APACHE-score, coronary artery disease, immunosuppression, ICU care duration in days, need for mechanical ventilation, vasopressor requirement and baseline eGFR. Furthermore, the intake of chloride was independently associated with eGFR at 90 days follow-up in multivariable linear regression model after further adjusting for baseline chloride (β = 0.215, CI 95% 0.026–0.119, *p* = 0.003).

Altogether the first, second, third and fourth quarter of the study time period comprised 124 (26.3%), 142 (30.1%), 111 (23.6%) and 94 (20.0%) patients, respectively. The median daily intakes of chloride during ICU care during the first, second, third and fourth quarter of the study period were 212 (132–305), 194 (128–304), 206 (129–352) and 156 (99–267) mmol, respectively. Similarly, the median daily intakes of sodium and calcium during ICU care were 355 (255–491) and 1.82 (0.92–2.76), 366 (251–535) and 2.59 (1.50–4.07), 401 (286–648) and 1.73 (0.37–3.07) and 296 (241–424) and 1.50 (0.58–2.47) for the first, second, third and fourth quarter of the study period, respectively. The differences in the daily intakes of chloride and sodium between the third and fourth quarter of the study period were significant (*p* = 0.029 and *p* = 0.002, respectively). The difference in the daily intake of calcium between the second and the fourth study quarter was significant (*p* < 0.001). The other pairwise comparisons for the daily intakes of chloride, sodium or calcium between study quarters were not. Hospital mortality decreased during the study period from 62 (50%) during the first quarter to 27 (28.7%) during the fourth quarter. The intake of chloride (R-square 0.223), sodium (R-square 0.259), calcium (R-square 0.155) and study time (R-square 0.028) was associated with hospital mortality in univariate logistic regression models (*p* < 0.01 for all comparisons). Moreover, the daily intake of chloride (odds ratio (OR) 1.005, CI 95% 1.003–1.005, *p* < 0.001) and study time (OR 0.757, CI 95% 0.625–0.916, *p* = 0.008) were independently associated with hospital mortality in a multivariable logistic regression model, as were the daily intakes of sodium (OR 1.004, CI 95% 1.003–1.005, *p* < 0.001) and calcium (OR 1.331, CI 95% 1.212–1.463, *p* < 0.001) in similarly adjusted separate multivariable logistic regression analyses.

## 4. Discussion

The cumulative daily intakes of chloride, sodium and calcium were independently associated with all-cause mortality within one-year and eGFR at 90 days follow-up after substantial adjustments in this large real-world retrospective cohort study on critically ill patients receiving CVVHD. However, total energy intake and the intake of protein or carbohydrates irrespective of the nutritional route were not associated with either outcome. While the intake of glucose and lipids was associated with mortality in the respective adjusted multivariable models, statistical significance for both covariates was lost in the separate sensitivity analyses excluding patients who died during the first three days of ICU care.

Our study is the first to demonstrate an association between cumulative daily chloride intake and mortality in critically ill AKI patients receiving CRRT. Furthermore, the association remained significant despite the substantial adjustment and exclusion of patients who died within the first three days. Notably, previous studies assessing the effect of chloride intake on patient and renal outcomes in critically ill patients have focused on those not receiving RRT at the study baseline [1,2,3,4,5,6,7]. Hospital mortality along with daily total chloride intake declined during the study period, and cumulative daily chloride intake was independently associated with hospital mortality in a multivariable logistic regression model adjusted for study time. Furthermore, the daily intake of chloride was a more significant explanatory variable for hospital mortality than the ICU admission time (study quarter) according to a higher R^2^ in univariate logistic regression analysis. Similar results were observed for the daily intake of sodium and calcium as well. Daily chloride intake was also associated with follow-up eGFR in survivors at 90 days follow-up. Although previous cohort studies comparing liberal and restrictive chloride regimens have shown similar results in relation to patient and renal survival outcomes [4,7], no definite differences in these outcomes have been demonstrated between resuscitation regimens of higher or lower chloride concentrations in RCTs or meta-analyses to date despite solid theoretical background to suggest otherwise [1,2,3,5,6,9]. Importantly, no RCT data exist on patient or renal outcomes in relation to chloride intake in critically ill AKI patients receiving CRRT. The administration of normal saline has been shown to induce persistent hyperchloremia, deterioration of the acid–base balance, greater expansion of the extracellular fluid volume and reduced renal perfusion, surrogate markers for poor patient and renal outcomes, compared to those with balanced crystalloids [8,13,14,15]. It is plausible that the positive association between chloride intake and mortality in our study is not only related to the high resuscitation volumes but also the choice of resuscitation solution, as both influence the total daily intake of chloride.

Our results on the association between chloride intake and patient and renal outcomes suggest interesting clinical implications for critically ill AKI patients receiving CRRT. Although studies comparing fluid regimens of higher and lower chloride concentrations have been inconclusive in critically ill non-RRT patients, the choice of resuscitation solutions lower in chloride concentrations may yield favorable outcomes in patients already on CRRT. Hyperchloremia in critically ill patients has been associated with poor patient outcomes and impaired renal recovery in those receiving CRRT [14,16]. Favoring fluids with a lower chloride concentration may ameliorate renal perfusion and mitigate the development of congestion [8] both of which are relevant for recovery in CRRT patients. In line with this reasoning, the International Fluid academy guidelines recommend balanced crystalloids for resuscitation in critically ill patients [17].

Interestingly, the intake of water was associated with mortality and follow-up kidney function, but the association between water intake and mortality ceased to be significant in the sensitivity analysis excluding the patients who died during the first days of ICU care. This finding suggests that the patients with the poorest prognosis received high fluid volumes during initial care, but during the course of a longer treatment period, the significance of fluid intake diminishes. Indeed, fluid overload has been associated with poor patient outcomes in critically ill AKI patients in prior reports [18]. However, our earlier work has shown that a lower cumulative fluid net balance resulting from fluid administration minus fluid removal during dialysis, diuresis and other losses in critically ill CRRT patients is associated with improved survival, suggesting that effective fluid removal may outweigh the prognostic significance of fluid intake during the course of ICU care in patients receiving RRT [19]. Intuitively, fluid balance is what matters, not the volume of fluids administered.

Sodium intake was associated with mortality in the current study and has also been linked to ICU care-related hypernatremia and adverse patient outcomes in prior studies [20]. Furthermore, the daily intake of sodium was independently associated with hospital mortality in a multivariable logistic regression analysis adjusted with study time. The cumulative daily intakes of sodium and chloride changed through the study time in a similar manner, probably reflecting the switch from favoring saline to favoring balanced crystalloids, which both have a lower concentration of sodium and chloride. Increased calcium intake in the form of supplementation has been associated with improved patient outcomes in unselected critically ill patients as opposed to our results [21]. However, the patients in our study had renal failure and a low median ionized calcium at baseline. In line with our findings, hypocalcemia has been associated with increased disease severity and mortality in prior studies [22]. The calcium intake in our study included the calcium administrated to reverse the local citrate anticoagulation during CVVHD and may therefore reflect lower plasma calcium levels or patient-related citrate accumulation due to insufficient citrate metabolism. However, this finding should be verified in independent studies to ascertain causality.

Total daily intakes of glucose and lipids were associated with mortality, while protein and amino acid intakes or total energy intake, regardless of the route of administration, were not. None of the associations between nutritional covariates and mortality remained significant when the patients who died in the first days of ICU care were excluded. In a recent meta-analysis on nutritional RCTs, no survival benefit was observed for enteral over parenteral nutrition, while the rate of infectious complications and the duration of hospital stay were lower in patients receiving predominantly enteral nutrition [12]. Overall, there is an unmet need for specific nutritional research and guidelines for critically ill AKI patients receiving CRRT, as the current iterations of ESPEN guidelines on AKI and ICU patients focus merely on energy and protein intake, disregarding the role of glucose, carbohydrate and lipid intake [10,11].

### Limitations

This study was retrospective in nature and has all the inherent limitations of a cohort study, and firm causal conclusions from the findings cannot be made. However, the study sample size was considerable, data were meticulously collected and the associations persisted through substantial statistical adjustments. Specific patient data on the solutions used or the timing of administered electrolyte concentrates in relation to the intake of resuscitation solutions during the study period were not available, and the resuscitation protocols were left to the discretion of the attending clinicians. Furthermore, it was not possible to compare the use of different commercial fluid preparations in this real-world cohort study as many different fluids and added electrolyte concentrates were administered at different time intervals during ICU care. However, up-to-date resuscitation and nutrition guidelines were implemented, and all cumulative solute, nutrition and fluid measures were carefully extracted from the ICU software yielding good quality real-world data and depicting accurately the administered daily solutes throughout the study period.

## 5. Conclusions

The cumulative intake of chloride, sodium and calcium is associated with all-cause one-year mortality in critically ill AKI patients receiving CRRT. Furthermore, chloride, sodium, calcium and water intake are associated with eGFR at 90 days follow-up.

Resuscitation solutions of lower chloride and sodium concentrations may be associated with improved patient and renal outcomes in critically ill CRRT patients. The optimal solute concentrations of resuscitation solutions for AKI patients requiring CRRT in the ICU setting require further RCT research.

## Figures and Tables

**Table 1:** Baseline characteristics and clinical outcomes.

Covariate	Value
Age (years)	66 (58–74)
Female	138 (29.3)
Male	333 (70.7)
Weight (kg)	85 (72–99)
Baseline eGFR (mL/min/1.73 m^2^) ^a^	72.2 (46.2–95.6)
Hypertension	304 (64.5)
Diabetes	189 (40.1)
Congestive heart failure	113 (24.0)
Atrial fibrillation	116 (24.6)
Coronary artery disease	134 (28.5)
Peripheral artery disease	60 (12.7)
Solid organ malignancy	35 (7.4)
Immunosupression	61 (13.0)
Mechanical ventilation	348 (73.9)
Vasoactive support	441 (93.6)
ICU care duration (days)	7 (3–13)
SOFA-score	10 (7–12)
APACHE-score	25 (21–30)
eGFR at 90 days follow-up (mL/min/1.73 m^2^) ^b^	55.6 (37.1–86.6)
Death within 1 year follow-up	251 (53.3)

^a^ Data missing in 75 (15.9) patients. ^b^ Data missing in 61 (24.4%) patients out of the 250 survivors at 90 days follow-up. eGFR = estimated glomerular filtration rate; ICU = intensive care unit; SOFA = Sequential organ failure assessment score; APACHE = Acute Physiology and Chronic Health Evaluation II score, IQR = inter-quartile range. Categorical values in parentheses are % values unless stated otherwise. Continuous variables are expressed as the median (IQR), as all continuous variables had a skewed distribution. Baseline eGFR was defined as the most recent measurement of eGFR within one year of admission.

**Table 2:** Baseline biochemical and daily cumulative solute, nutrition and fluid data.

Covariate (Baseline)	Value	Covariate (Cumulative)	Value
Potassium (mmol/L)	4.4 (3.9–4.9)	Potassium (mmol)	30 (19–53)
Chloride (mmol/L)	107 (102–110)	Chloride (mmol)	192 (125–304)
Sodium (mmol/L)	136 (132–139)	Sodium (mmol)	352 (255–506)
Magnesium (mmol/L) ^a^	0.79 (0.72–0.93)	Magnesium (mmol)	4 (2–5)
Phosphate (mmo/L) ^b^	1.48 (1.00–2.10)	Phosphate (mmol)	12 (5–17)
Calcium ionized (mmol/L)	1.05 (0.99–1.12)	Calcium (mmol)	2 (1–3)
		Glucose (g)	134 (93–166)
		Amino acids (g)	0 (0–28)
		Protein (g)	0 (0–6)
		Lipids (g)	8 (0–26)
		Water (ml)	3828 (3131–4943)
		Enteral carbohydrates (kcal)	603 (390–910)
		Enteral lipids (kcal)	6 (0–54)
		Total enteral energy intake (kcal)	104 (42–226)
		Total parenteral energy intake (kcal)	508 (297–814)
		Total energy intake (kcal)	691 (414–1060)

^a^ Data missing in 314 (66.7%) patients. ^b^ Data missing in 267 (56.7%) patients. ICU = intensive care unit, IQR = inter-quartile range. Categorical values in parentheses are % values unless stated otherwise. Continuous variables are expressed as the median (IQR) as all continuous variables had a skewed distribution.

**Table 3:** Univariate cox proportional hazards model for one-year mortality (left) and univariate linear regression model for eGFR at 90 days follow-up in survivors with available eGFR data (right).

Covariate	HR	CI 95%	*p*	Beta	CI 95%	*p*
Potassium (mmol)	0.993	0.987–1.000	0.043	0.206	0.131–0.704	0.005
Chloride (mmol)	1.003	1.002–1.003	<0.001	0.293	0.053–0.148	<0.001
Sodium (mmol)	1.002	1.002–1.002	<0.001	0.396	0.068–0.137	<0.001
Magnesium (mmol)	1.056	1.018–1.094	0.003	0.182	0.586–4.731	0.012
Phosphate (mmol)	0.949	0.934–0.964	<0.001	0.122	−0.099–1.233	0.122
Calcium (mmol)	1.159	1.131–1.187	<0.001	0.291	3.866–10.829	<0.001
Glucose (g)	1.002	1.000–1.004	0.047	0.118	−0.016–0.160	0.107
Amino acids (g)	0.994	0.987–1.000	0.037	0.070	−0.129–0.375	0.338
Protein (g)	0.980	0.965–0.994	0.006	0.109	−0.109–0.804	0.134
Lipids (g)	0.985	0.977–0.993	<0.001	0.085	−0.125–0.485	0.245
Water (ml)	1.000	1.000–1.000	<0.001	0.487	0.013–0.022	<0.001
Enteral carbohydrates (kcal)	1.000	1.000–1.000	0.751	0.095	−0.005–0.023	0.194
Enteral lipids (kcal)	0.998	0.997–1.000	0.025	0.125	−0.006–0.097	0.085
Total enteral energy intake (kcal)	1.000	1.000–1.000	0.860	0.068	−0.010–0.027	0.350
Total parenteral energy intake (kcal)	1.000	0.999–1.000	0.171	0.082	−0.006–0.022	0.260
Total energy intake (kcal)	1.002	1.000–1.000	0.286	0.110	−0.003–0.020	0.133

eGFR = estimated glomerular filtration rate; HR = hazard ratio; CI = confidence interval.

## Data Availability

The datasets used and/or analyzed during the current study are available from the corresponding author upon reasonable request.
